# The evolutionary dynamics of metabolic protocells

**DOI:** 10.1371/journal.pcbi.1006265

**Published:** 2018-07-20

**Authors:** Ximo Pechuan, Raymond Puzio, Aviv Bergman

**Affiliations:** 1 Department of Systems and Computational Biology, Albert Einstein College of Medicine, Bronx, New York, United States of America; 2 Dominick P. Purpura Department of Neuroscience, Albert Einstein College of Medicine, Bronx, New York, United States of America; 3 Department of Pathology, Albert Einstein College of Medicine, Bronx, New York, United States of America; 4 Santa Fe Institute, 1399 Hyde Park Road, Santa Fe, New Mexico, United States of America; CPERI, GREECE

## Abstract

Protocell multilevel selection models have been proposed to study the evolutionary dynamics of vesicles encapsulating a set of replicating, competing and mutating sequences. The frequency of the different sequence types determines protocell survival through a fitness function. One of the defining features of these models is the genetic load generated when the protocell divides and its sequences are assorted between the offspring vesicles. However, these stochastic assortment effects disappear when the redundancy of each sequence type is sufficiently high. The fitness dependence of the vesicle with its sequence content is usually defined without considering a realistic account on how the lower level dynamics would specify the protocell fitness. Here, we present a protocell model with a fitness function determined by the output flux of a simple metabolic network with the aim of understanding how the evolution of both kinetic and topological features of metabolism would have been constrained by the particularities of the protocell evolutionary dynamics. In our model, the sequences inside the vesicle are both the carriers of information and Michaelis-Menten catalysts exhibiting saturation. We found that the saturation of the catalysts controlling the metabolic fluxes, achievable by modifying the kinetic or stoichiometric parameters, provides a mechanism to ameliorate the assortment load by increasing the redundancy of the catalytic sequences required to achieve the maximum flux. Regarding the network architecture, we conclude that combinations of parallel network motifs and bimolecular catalysts are a robust way to increase the complexity of the metabolism enclosed by the protocell.

## Author summary

The protocell hypothesis conjectures the existence of a vesicle containing catalytic and replicating sequences as the primordial cellular organization during the early stages of the evolution of life. Mathematical models of protocells traditionally consider RNA sequences being encapsulated and having both an informational and a catalytic role in the same molecule. Because of this dual function, the protocell sequences are evolutionarily constrained. Mathematical models have been extensively used to study the evolutionary dynamics of protocells with a focus on the processes, like mutation or stochastic sequence assortment upon division, that affect the protocell information capacity in terms of the coexistence of different sequence types. Here we introduce a simple model of metabolic networks whose output determines the survival of the protocell with the aim of studying the effect of modifying the kinetic and architectural properties of the network on sequence coexistence. We find that stochastic assortment and mutation limit the architectures able to be encapsulated by the protocell with a given fraction of the population harbouring all possible sequence types.

## Introduction

The RNA world hypothesis [1–3] describes a stage in the early evolution of life where RNA constituted both the catalytic and the informational component of living systems. This scenario has a reasonable degree of plausibility accounted for by the evidence gathered from both prebiotic chemistry [4, 5] and the analytic approach of evolutionary biology [6] to the study of the origins of life. Constrained by this assumption, elucidating the evolutionary dynamics of primordial replicators becomes a fundamental question, initially addressed by the quasi-species model [7, 8]. This formalism assumed that error rates for early self-replicators must have been inherently high leading to a distinct evolutionary behavior, namely that a sequence, regardless of its fitness, never fixates in the population, but instead is always surrounded by its mutant neighborhood forming a quasispecies. Furthermore, given a sharply peaked fitness landscape, if the mutation rate is increased to a critical value, known as the error threshold, the extinction of the population known as the error catastrophe will occur. One possible solution would be to reduce the mutation rate, but that would almost certainly involve a more complex self-replicator, encoded by a sequence of considerable length, which is in turn limited by the error rate. This evolutionary *cul-de-sac*, known as Eigen’s paradox, required a reassessment of the model of protobiotic systems in light of their poor evolutionary potential. Trying to address this issue led to the consideration of distributing the information encoded by the sequences in a mutualistic community of replicators known as the hypercycle model [9] in order to relax the error rate. However, the large amplitude of the oscillations of the stationary state of the hypercycle model could lead to its extinction upon stochastic perturbation which, together with the susceptibility of the hypercycle model to the invasion of selfish molecules, make it difficult for the community of replicators to emerge without a form of population viscosity [10]. Viscosity could have been provided by limited spatial dispersal over a substrate like clay [11, 12] or as a limiting case, the compartmentalization of the sequences in a protocell acquiring a multi-level selection [13, 14] structure when a the vesicle survival is coupled to its sequence content.

The hypothetical protocell organization has been extensively modelled in the past decades, from population genetics multilevel selection models to explicit biophysical accounts of chemical reactions that contribute to the formation of the vesicle membrane [15, 16]. These two avenues have remained relatively separated in the literature and the population genetic models have not fully explored the consequences of deriving the fitness function from realistic models of catalysed reaction networks that could have occurred inside the vesicle. On the other hand, the biophysically inspired models are mainly concerned with the coupling of chemical reactions to the division of the membrane but do not incorporate population genetic considerations such as mutation, recombination or variations in heredity mechanisms. The population genetics multilevel selection protocell models have their origins in the package model [17, 18] and the stochastic error corrector model [19, 20]. These models describe the evolutionary dynamics of a set of replicating sequences encapsulated in a protocell and consider the processes that such a system would have had to endure at the dawn of life such as random assortment of the sequences upon fission, mutations to non-functional sequence types and conflict between selection at the sequence level and the vesicle level. All of these processes suppose that the protocell population exhibits an average fitness lower than the maximum achievable which is commonly referred as genetic load. In these models, the protocell level fitness is commonly specified by a simple function, like the geometric average, of the frequencies of the sequence types contained in the vesicle. The dynamics at the sequence level inside the protocell is described by a replication-mutation process. Deleterious mutation is typically introduced into the system in the form of irreversible mutation to a defective type that does not directly affect the protocell fitness except by reducing the effective total number of functional sequences. These protocell models account, in a non-systematic manner, for the study of additional complexities and processes to the protocell dynamics. For example, the incorporation of mutation schemes exhibiting trade-offs in catalytic activities [21, 22], modifications in the mechanism of inheritance or the incorporation of protocell fusion [23] and recombination [24]. Few of them have directly considered a model of the structure of the underlying metabolic network and those that have, did so in a limited manner by studying a linear chain of unsaturated Michaelis-Menten enzymes [25]. Most of the vesicle models depict a scenario where the nature of the encapsulated sequences is RNA, corresponding to the idea of the ribocell as a solid candidate for the protocellular stage in the early evolution of life [26].

The coupling between metabolism and replication would have been particularly strong in the hypothesized ribocells as the RNA sequences are supposed to have operated as both the catalysts and the carriers of the genetic information. We conjecture that this must have limited the space of possible catalysed chemical reaction networks which could be encapsulated within the protocells. In this work, we incorporate a simple implementation of enzymatic metabolism to the protocell model to address the problem of determining the complexity and architectural characteristics of early metabolic networks. The approach of considering the organism structure has been taken in other studies of the evolution of biological networks [27, 28] that highlight the relevance of accounting for some level of biological detail to study evolutionary dynamics.

Metabolic networks are modelled with different degrees of dynamic and kinetic detail. With a high level of kinetic detail, metabolic networks can be modelled with systems of differential equations incorporating aspects of the reaction mechanisms and properties of their catalysts. These same reactions can be studied in the stochastic limit when the number of particles considered is low. A framework that connects both the stochastic and the deterministic limits of a reaction network is that of chemical reaction network theory [29]. With a lower level of kinetic detail, one can use modelling techniques such as flux balance analysis to understand the general behavior of reaction fluxes at stationary states [30]. As a compromise between realism and computability we modeled the protocellular metabolism as a mass action irreversible reaction network with a small number of catalysts. In our model, each of the metabolic reactions is catalyzed by a sequence of a given type with a Michaelis-Menten kinetic mechanism and thus exhibiting saturation behavior. The output of this metabolic network determines the fitness of the protocell. We are interested in the transition to the coexistence of all sequence types at the protocell population level and how this depends on the kinetics and architecture of the network. For simple protocell models, the transition to coexistence has been extensively studied analytically [31]. Sequence coexistence provides a way to increase the complexity of protocellular metabolism by harboring different types of catalysts. We find that saturated metabolic fluxes provide a way to increase the coexistence of sequence types in the protocell by allowing the necessary redundancy to alleviate the fitness load produced by assortment when the protocell divides. We also find that the combination of parallel fluxes with a terminal bimolecular catalyst provides a way of increasing the complexity of the protocellular metabolism.

## Results

We model the protocell evolutionary dynamics as a Wright-Fisher process following the approach in [32]. We augment the package model with a fitness function given by the flux of a critical metabolite, *ϕ*(*M*), coming from a small metabolic network comprised of a set of chemical reactions, *r*, catalysed by the sequences inside the protocell. Each protocell contains a number of sequences from a finite set of sequence types, or genes, *g*, of cardinality |*g*| = |*r*| + 1, meaning that there is a sequence type catalysing each reaction of the metabolic network plus a mutational sink, the type *ω*, that does not contribute to the metabolic activity. The population dynamics is derived from the following protocell life-cycle. At the beginning of the life-cycle, *N* sequences are found in the vesicle distributed amongst the set of sequence types and each sequence is equally likely to replicate. The total number of sequences at the beginning of the protocell life-cycle will be herein referred as the protocell ploidy. When the sequences replicate they can mutate to the defective type, *ω*, with a probability traditionally unpacked in terms of the sequence length, *L*, and the mutation probability per base, *μ*, so that the accuracy of the replication process is given by *q* = (1 − *μ*)^*L*^. Hence, the sequences have a probability (1 − *q*) to mutate to the defective type. After reaching twice the ploidy, 2*N*, the vesicle splits in half and the sequences randomly assort between the offspring protocells regaining the starting total number of sequences *N*. At the population level, once all the protocells have divided, half of them are selected with a survival probability proportional to the critical metabolite flux. A simplified treatment of metabolic networks is used to define the protocell fitness function in our simulations. We evaluated the consequences on the protocell evolutionary dynamics of varying the kinetic parameters and the organization of the catalysts in different network topologies. Our model assumes the separation of the dynamics at three time scales. The quickest time scale is that of metabolic reactions catalysed by the sequences. The next time scale is that of the replication of sequences and division of the protocell. The third and longest time scale is that of the dynamics of the population of protocells. We make use of simplifying assumptions based on each of the time scales being much longer than the previous time scale.

**Fig 1:**
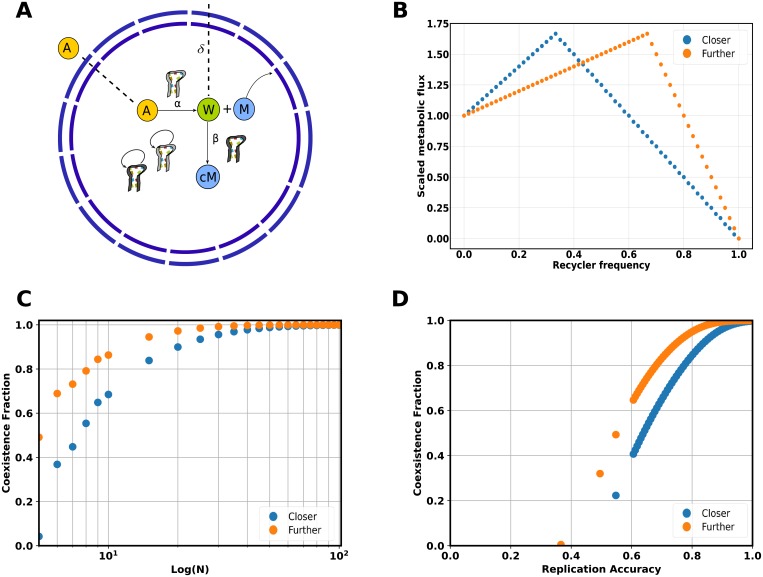
Coexistence fraction dependence on kinetic parameters for a simple two catalyst model. **A**: Depiction of a protocell encapsulating a simple keystone-recycler metabolic network. The first catalytic sequence type, the keystone (*α*) is essential for the production of the critical metabolite *M* from the substrate *A* that diffuses from the environment into the vesicle. The second sequence type, the recycler (*β*), catalyzes the transformation the waste product of the first reaction, *W*, to form $c_{M}^{\beta }$ molecules of *M*. The molecules of *M* are immediately utilized by the protocell and the intermediates can diffuse out through the vesicle membrane with permeability *δ*. **B**: The critical metabolite flux as a function of the frequency of the recycler sequence type *β* for a constrained total number of sequences in the protocell. The flux closer to the boundary corresponds to a catalyst *β* with kinetic parameters $k_{\text{cat}}^{\beta }=2$, $c_{M}^{\beta }=1.5$ and the flux further to the boundary considers the production of two waste molecules, $c_{w}^{\alpha }=2$, by the reaction catalyzed by *α*. **C**: The coexistence fraction for these two cases as the protocell ploidy is varied. **D**: For a fixed ploidy of *N* = 60, the decay of the coexistence fraction as a function of the replication accuracy for the two different fluxes.

We illustrate the effects of the variations in the kinetic and stoichiometric parameters of the network with the example of a protocell containing two catalysts (Fig 1A). This network is the starting point for the construction of larger networks which allow us study the effects of the network architecture on the protocell evolutionary dynamics. The first catalyst, the keystone (denoted as *α*), takes an environmental input *A* that diffuses inside the protocell to produce the critical metabolite, *M*, that is required for the protocell survival. The keystone catalyst also produces certain amount, $c_{w}^{\alpha }$, of a waste product, *W* that can be converted by a second catalyst, the recycler (denoted as *β*), into $c_{M}^{\beta }$ molecules of critical metabolite. A protocell composed solely of keystone catalysts is viable and thus constitutes an absorbing boundary of the stochastic process. For all of the networks studied, we assume that the environmental input is present at sufficiently large quantities such that its concentration is not affected by the protocell population growth and the keystone catalysts that use it are in the saturation regime. The corresponding metabolic flux for this scenario according to our formalism is given by (see Methods, section 4.1.2):

$$\begin{array}{c} \Phi =k_{\text{cat}}^{\alpha }\left[\alpha \right]+c_{M}^{\beta }\min\left(c_{w}^{\alpha }k_{\text{cat}}^{\alpha }\left[\alpha \right],k_{\text{cat}}^{\beta }\left[\beta \right]\right) \end{array}$$ (1)

Here *k*_cat_ denotes the catalytic constant of the catalysts. With the total ploidy constraint, the fitness function defined as the critical metabolite flux of this metabolic network (Fig 1B) will have a single maximum with a position on the space of protocell types given by the kinetic parameters and the protocell composition, more concretely when $c_{w}^{\alpha }k_{\text{cat}}^{\alpha }\left[\alpha \right]=k_{\text{cat}}^{\beta }\left[\beta \right]$. The position of the maximum determines the behaviour of the observables of the population like the coexistence fraction; the fraction of the population with protocells containing the maximum number of functional sequence types. This dependence can be shown by choosing the kinetic and stoichiometric parameters so that the flux is closer or further away from the absorbing boundary of protocells with no recycler sequences. One simple way of achieving this is by increasing the catalytic constant, $k_{\text{cat}}^{\beta }$, of the recycler sequence to get closer to the boundary or increasing the waste output of the keystone sequence by changing the stoichiometry, $c_{w}^{\alpha }$, to get further away from it. The remaining stoichiometric coefficients can be adjusted to achieve the same maximum flux for comparison as the fitness difference between the maximum in the interior and the fitness of the absorbing boundary of the stochastic process (that is when the protocell lacks any recycler sequences and just harbors the keystone) also affects the ploidy at which the coexistence transition occurs.

The coexistence fraction of the stationary population for the flux with the maximum further away from the absorbing boundary is always higher than the one closer to it (Fig 1C). This is due to the effects of stochastic assortment when protocells divide that can bring a fraction of the population to fixate at the boundary. This probability is smaller as we get further away from the boundary and it vanishes upon increasing the ploidy of the protocell leading to a stationary population of coexisting types only. Hence, fluxes with a maximum further away from the absorbing boundary benefit coexistence as they are less affected by the loss introduced by the assortment. In terms of the average fitness of the stationary population, the flux further away from the boundary also presents a higher average fitness for all ploidy values and consequently if the two fluxes were to be competed with no interaction between the protocells the flux further from the boundary would dominate at the infinite population size limit (see S1 Fig). This is because the flux that is further away from the boundary is also flatter than the other flux and thus suffers from less assortment load as the population can spread over a larger region of viable coexisting types.

When mutations leading to the defective type are taken into account, the decay of the coexistence fraction of the flux closer to the boundary is faster when increasing the mutation rate than that of the flux further away from it (Fig 1D). In terms of average fitness of the populations, both landscapes show similar values at low mutation rates but the flux further to the boundary becomes fitter as the mutation rate increases and the coexisting types are lost in the flux closer to the boundary. As noted by [32], the way mutation towards a defective sequence type is usually included in the protocell model has no other effect at the protocell level than reducing the total number of functional types in the vesicle. Therefore, the impact of mutation on the coexistence fraction of the stationary population will be equivalent to that of reducing the ploidy to the effective ploidy given the mutation rate and the starting ploidy of the protocell. For a given flux function and mutation rate, the effective ploidy will saturate at a constant value as the total starting ploidy is increased. At high mutation rates the effective ploidy becomes sensitive to the assortment in the flux closer to the boundary and thus the flux further away from the boundary is also more robust to the effects of introducing non-functional mutants.

**Fig 2:**
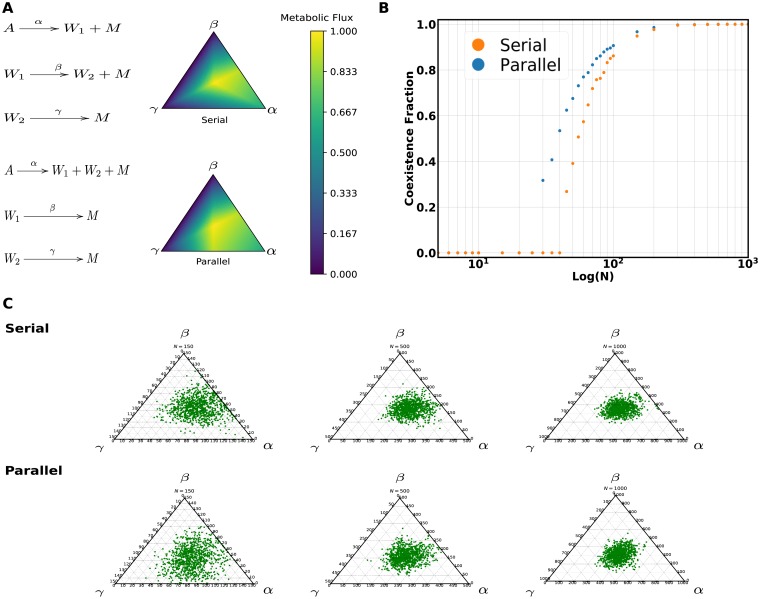
Coexistence fraction variation with ploidy for the two studied three catalyst architectures. **A**: The two catalyst architectures and their respective normalized value of the critical metabolic flux over the space of protocell types. **B**: The dependence of the coexistence fraction with the protocell ploidy for the serial and parallel architectures. The kinetic and stoichiometric parameters of the flux function were chosen for each architecture to have the same maximum flux and marginal benefit of the third catalyst (in this case all catalytic constants were set to one and $c_{M}^{\beta }=c_{M}^{\gamma }=4$). **C**: Ternary plots of the stationary distribution from populations with different ploidy values (N of 150, 500, 1000) for each architecture.

We now focus on analysing effects of network architecture by considering two different arrangements of three catalytic sequences (Fig 2A). We will add the third recycler catalyst in serial or parallel with respect to the second recycler sequence of the previous example. In both cases studied, we assume for simplicity that the keystone catalyst produces two different waste products to have non-competing reactions. In order to discern effects derived from the topology of the networks alone, the total fitness benefit a protocell containing all three catalysts and the marginal fitness benefit of the third sequence with respect to the other two given by the kinetic and stoichiometric parameters was set to be the same for each of the studied architectures. According to our simplifying treatment (see Supplement, section 7.1), the output metabolite fluxes for these two architectures are:

$$\begin{array}{c} \Phi_{Serial}=k_{\text{cat}}^{\alpha }\left[\alpha \right]+c_{M}^{\beta }\min\left(k_{\text{cat}}^{\alpha }\left[\alpha \right],k_{\text{cat}}^{\beta }\left[\beta \right]\right)+c_{M}^{\gamma }\min\left(k_{\text{cat}}^{\alpha }\left[\alpha \right],k_{\text{cat}}^{\beta }\left[\beta \right],k_{\text{cat}}^{\gamma }\left[\gamma \right]\right) \end{array}$$ (2)

$$\begin{array}{c} \Phi_{Parallel}=k_{\text{cat}}^{\alpha }\left[\alpha \right]+c_{M}^{\beta }\min\left(k_{\text{cat}}^{\alpha }\left[\alpha \right],k_{\text{cat}}^{\beta }\left[\beta \right]\right)+c_{M}^{\gamma }\min\left(k_{\text{cat}}^{\alpha }\left[\alpha \right],k_{\text{cat}}^{\gamma }\left[\gamma \right]\right) \end{array}$$ (3)

The fluxes over the simplex of protocell types all have their maxima at the center for the parameters chosen removing the effect of the proximity to the absorbing boundaries. The transition to coexistence for the parallel addition of the third catalyst occurs at a lower ploidy values than that of the serial addition (Fig 2B). The coexistence fraction also grows faster with ploidy for the parallel architecture. Both observations can be explained in terms of the assortment load, as the parallel architecture has a larger region of viable protocell types surrounding the maximum making it more robust to assortment by allowing the population to spread over this region (Fig 2C). The serial architecture, on the other hand, has a smaller region of viable types surrounding the maximum and becomes more sensitive to the assortment. The mean fitness of the population reflects also this pattern given by the assortment load (S2 Fig). It can be concluded that for our model the population observables are mostly determined by the features of the fitness landscape given by the metabolic flux. The protocell population can be seen as a quasispecies with a cloud of vesicles around the fittest type spread by the assortment at a particular ploidy. Adding mutation to the system does not change the conclusion of the parallel addition higher robustness as mutations factor in the dynamics by effectively reducing the ploidy of the system.

The process of increasing the complexity of the studied networks by sequentially connecting to them additional recycler catalysts given the considered motifs, raises the question about what is the best way to perform this addition in order to guarantee a transition to coexistence of the resulting networks at low ploidy values. Adding a new sequence type to the protocell always comes at the cost of increasing the effects of the assortment, which depends on ratio the protocell ploidy and the total number of sequence types. This means that any increase in the coding capacity of the protocell, in terms of the number of coexisting sequence types, requires in turn an increase in the vesicle ploidy. It has been shown that by making the ploidy sufficiently large, the numbers of types that can coexist in the protocell can be made to increase arbitrarily [32, 33]. This is, however, just for the cases with low competition at the sequence level and no mutations. The requirement of increasing the ploidy becomes a burden once mutation is considered, as higher ploidy protocells accumulate mutants at a faster rate (S3 Fig). If certain amount of mutants are considered deleterious as in the early versions of the package model, the mutation process will set a limit to the ploidy increase. The mutation process also establishes a limit on the number of catalytically active sequences in the protocell by saturating to a constant effective ploidy regardless of the starting ploidy value. This can bring the population to the absorbing boundaries if the effective ploidy is low enough for the assortment to dominate. A way of keeping the ploidy moderate while adding new catalysts to the network is by increasing the marginal fitness benefit of each added sequence (changing the stoichiometric coefficient *c*_*M*_). This can substantially lower the critical ploidy for the coexistence transition and its effects will depend on both the fitness benefit and the topology of the previous architecture in place. For instance, if we were to add another catalyst with a high marginal fitness benefit to our two previous examples, the parallel architecture would become unbalanced leading to the fixation of one of the branches while the serial addition would still be viable. In order for the parallel architecture to accept the new sequence with a higher marginal fitness benefit without becoming unbalanced a bimolecular catalyst should be considered.

A bimolecular catalyst can be added to two independent keystone catalysts and allow them to coexist. We consider the case of two keystone catalysts converting two independent environment metabolites into the critical metabolite and also producing two different waste products that are in turn required for the third recycler catalyst in a bimolecular reaction to produce more metabolite of interest (Supplement, section 7.2. The parameters were chosen to have the same marginal fitness benefit and maximum fitness than the previously studied cases. According to our simplifying the flux would be:

$$\begin{array}{c} \Phi_{Bimolecular}=c_{M}^{\alpha }k_{\text{cat}}^{\alpha }\left[\alpha \right]+c_{M}^{\beta }k_{\text{cat}}^{\beta }\left[\beta \right]+c_{M}^{\gamma }\text{min}(k_{\text{cat}}^{\alpha }\left[\alpha \right],k_{\text{cat}}^{\beta }\left[\beta \right],k_{\text{cat}}^{\gamma }\left[\gamma \right]) \end{array}$$ (4)

**Fig 3:**
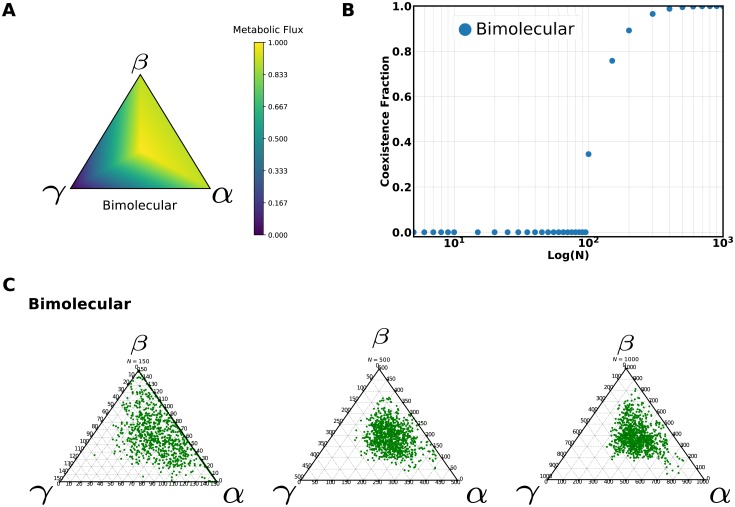
Coexistence fraction variation with ploidy for the bimolecular architecture. **A**: The value of the critical metabolite flux over the space of protocell types. **B**: The coexistence fraction variation for this architecture and its dependence on ploidy. **C**: Ternary plots of the stationary distribution from populations with different ploidy values (N of 150, 500, 1000). All catalytic constants were set to one and $c_{M}^{\beta }=c_{M}^{\alpha }=2.5$ and $c_{M}^{\gamma }=4$).

The bimolecular motif flux exhibits a large region of viable types around the maximum however, the protocell population is also able to spread to an absorbing boundary with a large neutral space as the two keystone catalyst have the same marginal fitness benefit (Fig 3A). These features of the fitness landscape contribute to a large assortment load and a substantial fraction of the population is close to the absorbing boundary even at relatively high ploidy values (Fig 3C). Therefore, the coexistence transition for this architecture occurs at larger ploidy values than the other two architectures in spite of a larger region of viable types surrounding the flux maximum (Fig 3B). On the other hand, the third bimolecular catalyst is able to provide a way for the coexistence of these two keystone catalysts. Moreover, the bimolecular reaction gives us a way of adding a catalyst with a large fitness benefit to the parallel architecture without unbalancing it.

**Fig 4:**
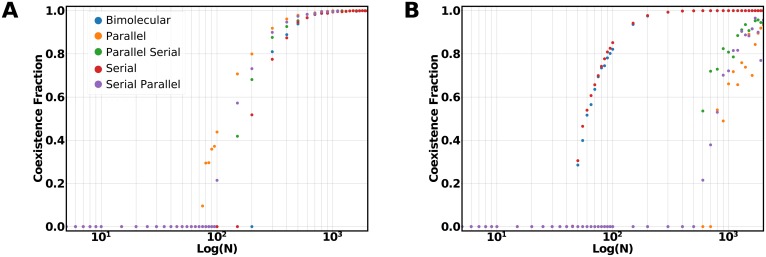
Coexistence fraction variation with ploidy for the four catalysts architectures. **A**: Coexistence fraction behavior considering equal stoichiometric production of the metabolite of interest for all the recycler catalysts in the network $c_{M}^{\beta }=c_{M}^{\gamma }=c_{M}^{\theta }=6$. **B**: Coexistence fraction behavior when the addition catalyst *θ* has a higher benefit $c_{M}^{\theta }=10$.

We now consider the fluxes derived from the addition of a fourth catalytic sequence to the previous networks as a unimolecular or bimolecular recycler (Supplement, section 7.3). We again consider the production of an additional waste metabolite to have non-competing reactions. When the added recycler catalyst has the same marginal fitness benefit as the other two recyclers, the parallel architecture shows the transition to coexistence at the lowest ploidy value, as in the three catalyst networks previously analysed (Fig 4A). The parallel architecture is followed by the two other architectures with parallel motifs showing that for the case of equal marginal fitness benefit the parallel architectures are better at supporting the coexistence of catalyst and thus maintaining metabolic complexity. This is due to the larger region of viable types surrounding the flux maximum for the parallel architectures. When the marginal fitness benefit of the fourth recycler catalyst is greater than the other two, the parallel architectures are no longer the ones showing coexistence transitions at lower ploidy values. The bimolecular and the serial architectures will be the ones showing a markedly earlier transition to coexistence than the architectures with parallel motifs (Fig 4B). The addition of a recycler with larger marginal fitness benefit unbalances the parallel architectures unless it is added as a bimolecular motif. Increasing the complexity of metabolic networks in the protocell model requires the balance between the limitations given assortment load, the marginal fitness benefit and the risk of unbalancing the previously existing architectures. We find that combining bimolecular and parallel pathways one can achieve networks with transitions to coexistence at lower ploidy values that would avert the problems introduced by mutations at large ploidy values. Our finding is interesting in the light of the prevalence of bow-tie modules in extant metabolic networks [34, 35] as the concatenation of the parallel and bimolecular motifs could produce such a network feature.

## Discussion

Mathematical models of early life forms constitute a powerful tool to test the feasibility of proposed hypothetical scenarios in the first stages of life. They allow us to increase our understanding of the conjectured scenarios and provide intuition about how to overcome their limitations. Our metabolic model of the protocell serves to analyse the effects of considering details of the kinetic mechanism of the catalysts encapsulated and their network architecture. Our model provides an example of how the fitness load introduced by protocell fission assortment process and its effects on the coexistence of catalysts can be alleviated by modifications in their kinetic parameters and their network organization. The requirement to withstand the assortment load limits at low ploidy values the metabolic architectures that can be effectively encoded in the protocell. Although the networks analyzed here are simple and small, they already show interesting consequences of accounting for the saturation of the catalysts in different architectures. We demonstrate that variations in the kinetic parameters that keep the catalyst saturated provide a way to increase the catalyst redundancy and diminish the assortment effects. The saturation of the catalysts means that the reaction they control becomes limiting and additional catalytic sequences will consequently increase the output flux.

The other major limitation for the evolution of a complex protocellular metabolism is the interplay of the effects of the assortment and the network architecture. We demonstrate that architectures with a larger region of viable types surrounding the maximum in the interior favour the coexistence transition as they allow for the quasispecies to spread. The architectures that are favoured also depend on how they are assembled, more specifically, the marginal fitness benefit of the new catalyst with respect to the previous network catalysts. This benefit can happen directly by a higher stoichiometric benefit or indirectly by increasing the catalytic efficiency of the enzyme which allows the limiting catalyst to increase their numbers in the vesicle virtue of the flux saturation. For architectures containing parallel motifs it will be detrimental to accept the new catalyst if it has a higher marginal fitness benefit unless it is involved in a bimolecular reaction. The bimolecular motif prevents the system from becoming unbalanced and becomes a fundamental building block for larger architectures. Nevertheless, the assortment load together with the coupling of genes and catalysts, limit the architectures of the metabolic networks that can be maintained in the vesicle by coexistence of its catalysts. This limit in the metabolic evolutionary potential of the protocell can be alleviated, as it has been previously pointed out [36], by the evolution of the decoupling between genetic information and catalytic activity, either with the advent of proteins or DNA in a ribozyme world or by the divergence of complementary RNA sequences [37]. The evolution of correlated inheritance like the chromosomal organization would be another mechanism that would allow the exploration of a larger space of network architectures and catalytic efficiency [38] as it would diminish the assortment load. An example of releasing this constraint can be found in [25] which makes use of the unsaturated linear metabolic chain formulation of [39] to address the effects of chromosomal organization in the evolution toward specialization of promiscuous catalysts.

The model presented incorporates assumptions about the biochemistry of the hypothetical protocell and standard population dynamics that can be sophisticated or relaxed with interesting consequences. For example, the incorporation of trade-off between catalytic and replication activity has shown interesting dynamical features at the population level such as sustained oscillations [40]. The metabolism model could be further enriched in the direction of the whole cell model [41] by increasing the biochemical details considered. The only limitation is that the nature of early living things is purely conjectural and it would be preferable to understand general features of the evolution of metabolic networks rather than a concrete instantiation mirroring extant life. Together with this consideration, a better model for the development stage of the protocell and the corresponding population dynamics would allow us to decompose the summary statistic of the evolutionary dynamics, that is fitness, in terms of the whole interplay of the network flux and the replication of catalytic sequences throughout the developmental stage. A more detailed account on protocell development would require a full stochastic treatment that properly accounts for the separation of metabolic and replicative dynamic time scales. The effects of introducing other processes in the population dynamics like alternative division mechanisms in the metabolic protocell model as described by [42, 43] or modification in the heredity mechanisms already studied in previous protocell models could also yield interesting results. Further incorporating considerations about environment would lead us in the direction of understanding the ecology of the protocells. If our intermediates that leak out accumulate significantly in the environment [44], interesting frequency dependent relations can be established in the protocell populations and one could add protocell fusion to study the assembly of networks in this augmented model.

The protocell organization is considered a solid candidate for a transitional form of life and, as such, has been extensively explored computationally and is starting to be addressed in experimental settings [45]. The utility of the protocell theoretical models has been recently validated in helping the development of chemical implementations of protocell structures [46]. This also provides the experimental set-up to test the conclusions of the theoretical works in the protocell literature. Furthermore, construction of the first synthetic autonomous protocell is becoming a reality and we believe that theoretical models can be useful to further this endeavour.

## Methods

### Metabolic model

We will model the metabolic activity of our protocell as a well-stirred reactor in which ribozymes, environmental inputs, and metabolites interact. More specifically, the metabolic dynamics will be modelled as Michaelis-Menten enzyme dynamics where each flux is given by the expression

$$\begin{array}{c} v=\frac{k_{\text{cat}}\left[E\right]\left[S\right]}{K_{m}+\left[S\right]} \end{array}$$ (5)

Where, [*E*] and [*S*] denote the concentrations of enzyme and substrate respectively and *k*_cat_ is the turnover number (catalytic constant) of the enzyme and *K*_*m*_ its Michaelis constant. We will also study bimolecular reactions with mechanisms of the following form:

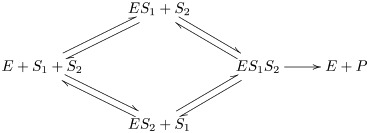
(6)
The velocity will have the functional form

$$\begin{array}{c} v_{r}=\frac{k_{\text{cat}}\left[S_{1}\right]\left[S_{2}\right]}{K_{m}+a_{1}c_{1}+a_{2}c_{2}+c_{1}c_{2}} \end{array}$$ (7)

where the values of *a*_1_ and *a*_2_ can be determined from either the forward and the backward rates of the underlying reactions or from thermodynamic potentials. Special values of these constants correspond to the cases of random order, definite order, non-interacting. We will model the depletion reactions as exponential decay or diffusion

$$\begin{array}{c} v_{\delta }=\delta_{i}\left[S\right] \end{array}$$ (8)

where *δ*_*i*_ is the decay rate or diffusion constant for *S*.

We assume that even though the protocell development will be accompanied by an increase in the vesicle volume, this will be negligible and will not dilute significantly the concentrations of the catalysts and intermediates of the reaction network. Therefore we can identify the catalysts’ concentrations with the catalyst number in our model of metabolism. We will augment this hypothesis with three further assumptions. We assume that the supply of this input in the environment is high enough that we can disregard depletion and treat its concentration as constant (but note that the concentration of the same inputs *within* the protocell may vary due to the permeability of the membrane). We assume that the intermediates that leak out to the environment are degraded and do not accumulate. We assume that the characteristic timescale of the metabolic reactions is sufficiently faster than the timescale of sequence replication that we may ignore initial transients, approximate the metabolic reactions by their (dynamic) equilibrium solution, and describe the metabolic outputs by their averaging their rates of production over a metabolic timescale.

#### Network motifs

Many interesting networks of biochemical interest can be built up by assembling copies of a few simple motifs [47]. We will now derive the steady state conditions on fluxes through two classes of motifs under the assumptions of irreversible Michaelis-Menten kinetics and small diffusion out the membrane. Because of the assumption of irreversibility, we may obtain the fluxes for a large class of networks by composing these conditions.

The first class of motifs consist of a unimolecular reaction in which an enzyme *E* reacts with one molecule of substrate *S* to produce one or more products *P*_*i*_:

$$\begin{array}{c} \begin{array}{cc} E+S & \to E+c_{1}P_{1}+c_{2}P_{2}+\cdots \\ S & \to \emptyset \end{array} \end{array}$$ (9)

Graphically, we may represent these motifs by the following diagram:

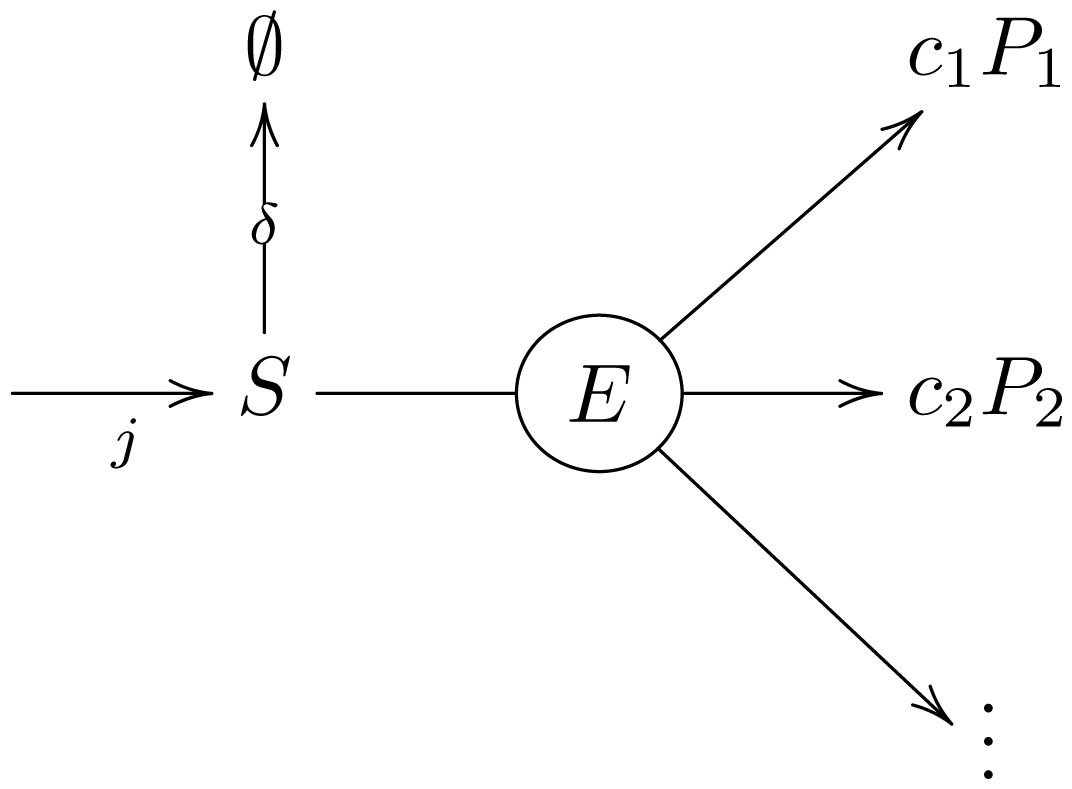
(10)
On account of irreverisbility, the velocity of the reaction does not depend upon the products or their stoichiometry. Letting *j* be the flux of substrate *S* and *δ* be the diffusion constant of the membrane, we have the following kinetic equations:

$$\begin{array}{c} \begin{array}{cc} j & =v+\delta [S]\\ v & =\frac{k_{\text{cat}}\left[E\right]\left[S\right]}{K_{m}+\left[S\right]} \end{array} \end{array}$$ (11)

Eliminating the concentration [*S*] between these equations produces an equation which can be solved to express the reaction velocity *v* as a function of the incoming flux *j*. To incorporate the assumption that the diffusion constant *δ* is small, we will expand this equation in powers of *δ*.

$$\begin{array}{c} \left(v-j\right)\left(v-k_{\text{cat}}\left[E\right]\right)+\delta K_{m}v=0 \end{array}$$ (12)

In the limit *δ* → 0, this equation reduces to (*v* − *j*)(*v* − *k*_cat_[*E*]) = 0, which has the solutions *v* = *j* and *v* = *k*_cat_[*E*]. The choice of solution depends upon the value of *j* and is determined by the conditions that the concentration is positive; thus, when *j* < *k*_cat_[*E*], we should choose the solution *v* = *j* and, when *j* ≥ *k*_cat_[*E*], we should choose *v* = *k*_cat_[*E*]. As a result, the zeroth order term in the expansion of the the velocity is a piecewise linear function of the incoming flux,

$$\begin{array}{c} v=\{\begin{array}{cc} j & j<k_{\text{cat}}\left[E\right]\\ k_{\text{cat}}\left[E\right] & j\geq k_{\text{cat}}\left[E\right] \end{array}, \end{array}$$ (13)

which may be expressed more succinctly as *v* = min(*j*, *k*_cat_[*E*]). The higher order terms can be regarded as corrections which account for deviations from piecewise linearity. Most notably, they replace the sharp peak at the maximum with a smooth peak whose maximum value is lower.

The second class of motifs consist of a bimolecular reaction in which an enzyme *E* reacts with molecule of substrate *S*_1_ and a molecule of substrate *S*_2_ to produce one or more products *P*_*i*_. This is described by the reactions

$$\begin{array}{c} \begin{array}{cc} E+S_{1}+S_{2} & \to E+c_{1}P_{1}+c_{2}P_{2}+\cdots \\ S_{1} & \to \emptyset \\ S_{2} & \to \emptyset . \end{array} \end{array}$$ (14)

the diagram

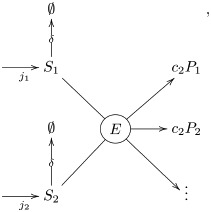
(15)
and the kinetic equations

$$\begin{array}{c} \begin{array}{cc} j_{1} & =v+\delta [S_{1}]\\ j_{2} & =v+\delta [S_{2}]\\ v & =\frac{k_{\text{cat}}\left[E\right]\left[S_{1}\right]\left[S_{2}\right]}{K_{m}+a_{1}\left[S_{1}\right]+a_{2}\left[S_{2}\right]+\left[S_{1}\right]\left[S_{2}\right]}. \end{array} \end{array}$$ (16)

Eliminating the concentrations [*S*_1_] and [*S*_2_] and collecting powers of *δ* leads to the equation

$$\begin{array}{c} \left(v-j_{1}\right)\left(v-j_{2}\right)\left(v-k_{\text{cat}}\left[E\right]\right)-\left(a_{1}v+a_{2}v-a_{1}j_{1}-a_{2}j_{2}\right)v\delta +K_{m}v\delta^{2}=0. \end{array}$$ (17)

In the limit *δ* → 0, this reduces to (*v* − *j*_1_)(*v* − *j*_2_)(*v* − *k*_cat_[*E*]) = 0. As previously, the choice of root is determined by the values of *j*_1_ and *j*_2_ through the condition that the concentrations [*S*_1_] and [*S*_2_] not be negative. This leads to a piecewise linear form of the leading term in the expansion of the solution,

$$\begin{array}{c} v=\{\begin{array}{cc} j_{1} & j_{2}\leq j_{1}<n_{E},V_{E}\\ j_{2} & j_{1}<j_{2}<n_{E}V_{E}\\ k_{\text{cat}}\left[E\right] & k_{\text{cat}}\left[E\right]\leq j_{1},j_{2} \end{array}, \end{array}$$ (18)

which may more conveniently be expressed in the form *v* = min(*j*_1_, *j*_2_, *k*_cat_[*E*]).

#### Two catalyst network: Keystone and recycler

As a simple illustration of how to use these motifs, consider a network consisting of an environmental input *A*, two enzymes *α* and *β*, an intermediate metabolite *W* and an output metabolite *M* and reactions

$$\begin{array}{c} \begin{array}{cc} A+\alpha & \to \alpha +W+M\\ W & \to \emptyset \\ W+\beta & \to \beta +c_{M}^{\beta }M \end{array} \end{array}$$ (19)

This may be represented by the diagram

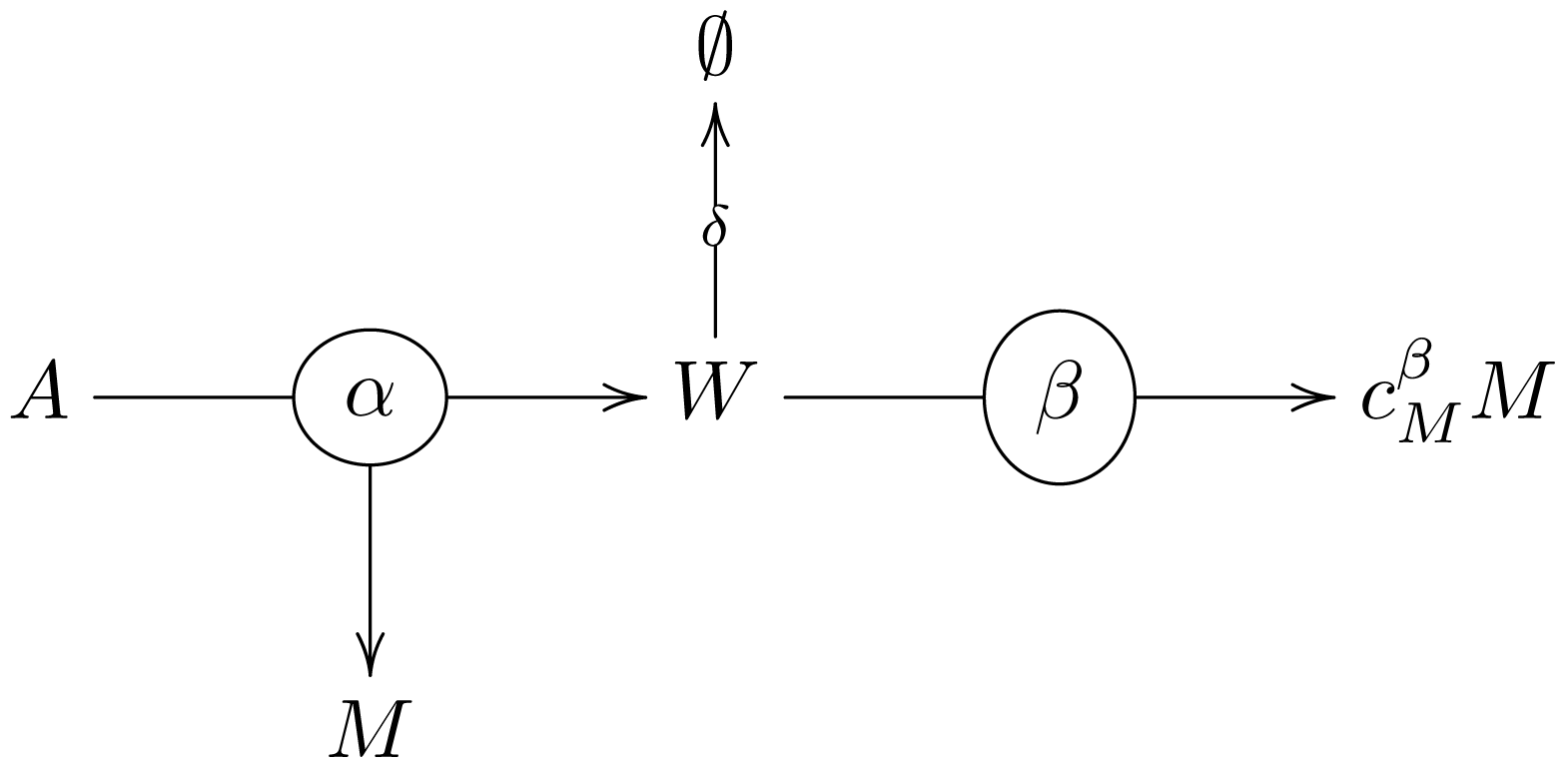
(20)
The velocity of the first reaction, which is catalyzed by *α*, referred in the text as the keystone, is given by the Michaelis-Menten formula. However, since we assume that the input *A* is plentiful in the environment, we expect this reaction to be happening well within the saturation regime, hence will approximate its velocity by the limiting velocity:

$$\begin{array}{c} v_{\alpha }=k_{\text{cat}}^{\alpha }\left[\alpha \right] \end{array}$$ (21)

The remaining two reactions, where *β* (referred in the text as the recycler) takes part are an instance of the first type of motif, so we have

$$\begin{array}{c} v_{\beta }=\min\left(j,k_{\text{cat}}^{\beta }\left[\beta \right]\right) \end{array}$$ (22)

Here *j* is the flux of *W*. Since the stochiometry of the first reaction produces one molecule of *W* for every molecule of input, we have *j* = *v*_*α*_, hence

$$\begin{array}{c} v_{\beta }=\min\left(v_{\alpha },k_{\text{cat}}^{\beta }\left[\beta \right]\right)=\min\left(k_{\text{cat}}^{\alpha }\left[\alpha \right],k_{\text{cat}}^{\beta }\left[\beta \right]\right). \end{array}$$ (23)

The total flux of *M* will include the output of both reactions:

$$\begin{array}{c} \Phi =v_{\alpha }+c_{M}^{\beta }v_{\beta }=k_{\text{cat}}^{\alpha }\left[\alpha \right]+c_{M}^{\beta }\min\left(k_{\text{cat}}^{\alpha }\left[\alpha \right],k_{\text{cat}}^{\beta }\left[\beta \right]\right). \end{array}$$ (24)

Using exactly the same techniques and assumptions as in the foregoing simple example, we calculated the flux-fitness functions for several more complicated networks involving three and four enzymes. The results of these calculations were then inserted into the evolutionary population model described below to study the effects of network architecture.

### Protocell development rate

Our aim is to enrich the evolutionary population genetic models with relevant biochemical features rather than go on the direction of a full-blown simulation of a cellular system. However, in order to introduce these features realistically, we will derive them from a simplified “whole protocell” model in which the metabolic reactions described above are augmented with reactions that describe the replication of the sequences:

$$\begin{array}{c} \begin{array}{cc} \alpha +M & \overset{}{\to }2\alpha \\ \beta +M & \overset{}{\to }2\beta \end{array} \end{array}$$ (25)

We will model the replication as mass action kinetics. As described in the previous section, we will model the enzymatic reactions using Michaelis-Menten kinetics. The only difference is that now we will augment the equation for the critical metabolite with a term which accounts for the depletion of metabolite as the sequences replicate.

$$\begin{array}{c} \frac{d[M]}{dt}=\Phi -r\left[M\right]\left(\left[\alpha \right]+\left[\beta \right]\right) \end{array}$$

For the two catalyst keystone recycler network, we have the system of equations:

$$\begin{array}{cc} \frac{d[\alpha ]}{dt} & =r[M][\alpha ]\\ \frac{d[\beta ]}{dt} & =r[M][\beta ]\\ \frac{d[M]}{dt} & =k_{\text{cat}}^{\alpha }\left[\alpha \right]\left[A\right]+c_{M}^{\beta }\frac{k_{\text{cat}}^{\beta }\left[\beta \right]\left[W\right]}{K_{m}^{\beta }+\left[W\right]}-r\left[M\right]\left(\left[\alpha \right]+\left[\beta \right]\right)\\ \frac{d[W]}{dt} & =k_{\text{cat}}^{\alpha }\left[\alpha \right]\left[A\right]-\delta \left[W\right]-\frac{k_{\text{cat}}^{\beta }\left[\beta \right]\left[W\right]}{K_{m}^{\beta }+\left[W\right]} \end{array}$$

For convenience, we introduce two rescaled variables—the ratio of types *x* and the scaled flux *ϕ*:

$$\begin{array}{cc} x & =\frac{\left[\alpha \right]}{\left[\alpha ]+[\beta \right]}\\ \phi & =\frac{\Phi }{\left[\alpha ]+[\beta \right]}=k_{\text{cat}}^{\alpha }x\left[A\right]+c_{M}^{\beta }\frac{k_{\text{cat}}^{\beta }\left(1-x\right)\left[W\right]}{K_{m}^{\beta }+\left[W\right]} \end{array}$$

Combining the first two equations, we conclude that

$$\begin{array}{cc} \frac{dx}{dt} & =\frac{\left(\left[\beta \right]\frac{d[\alpha ]}{dt}-\left[\alpha \right]\frac{d[\beta ]}{dt}\right)}{{\left(\left[\alpha \right]+\left[\beta \right]\right)}^{2}}\\ & =\frac{r[M]([\beta ][\alpha ]-[\alpha ][\beta ])}{{\left(\left[\alpha \right]+\left[\beta \right]\right)}^{2}}=0 \end{array}$$

so the ratio *α* to *β* remains constant during development. Since *ϕ* only depends upon *α* and *β* through their ratio, *ϕ* will also remain constant. As per the “slow-fast” minority control hypothesis of Kaneko and Yomo [48], we shall assume that the counts and reaction rates of the sequences are much smaller than the counts and reaction rates of the metabolites. In terms of the constants, this means that $k_{\text{cat}}^{\alpha },k_{\text{cat}}^{\beta }>>r$. As a consequence, the concentrations of *M* and *W* will approach an equilibrium values after an initial transient. Combining the equations, we conclude that

$$\begin{array}{c} \frac{d}{dt}\left(\left[\alpha \right]+\left[\beta \right]\right)=r\left[M\right]\left(\left[\alpha \right]+\left[\beta \right]\right)=\phi \left(\left[\alpha \right]+\left[\beta \right]\right). \end{array}$$

### Protocell evolutionary dynamics

The general protocell model for our study will be built following a similar approach to the package model [17, 32]. We will add to it an explicit representation of the metabolism by considering a set of chemical reactions, R, catalyzed by the sequences contained in the protocell. Each protocell will contain a number of sequences from a finite set of sequence types, *g*, that, in principle will be of cardinality |g|=|R|+1, indicating that there will be a sequence catalyzing each reaction, r∈R, of the metabolic network plus a mutational sink, the type *ω*, that will not contribute to the metabolic activity.

We will model the protocell development process as 2*N* independent samplings with replacement from the initial contents of the vesicle to represent the replication of sequences. Let *j*_*i*_ denote the number of number of sequences of type *i* present at the beginning of the cycle. The probability of sampling a sequence of type *i* equals *j*_*i*_/*N*. The probability that a replication will produce a sequence of non-error type *i* equals

$$\begin{array}{c} q\frac{j_{i}}{N} \end{array}$$ (26)

and the probability of producing an error type *ω* equals

$$\begin{array}{c} 1-q+q\frac{j_{\omega }}{N}. \end{array}$$ (27)

Hence the probability of an outcome in which there are *k*_*i*_ offspring of the sequence type *i* is given as

Pdev(ki|ji)=(2Nk1,k2,…kω)∏i=1g(qjiN)ki(1-q+qjωN)kω (28)

The probability that a protocell of this composition will then assort into an vesicle containing *m*_*i*_ sequences of type *i* and an offspring with *k*_*i*_ − *m*_*i*_ sequences of type *i* equals to

$$\begin{array}{c} P_{\text{asst}}(m_{i};k_{i}-m_{i}|k_{i})=\frac{\prod_{i=1}^{g}\left({}_{m_{i}}^{k_{i}}\right)}{\left({}_{N}^{2N}\right)} \end{array}$$ (29)

The probability of a particular outcome at the end of the lifecycle equals the product of the development and assortment probabilities. Defining $m_{i}^{\prime }=k_{i}-m_{i}$ and simplifying with the aid of binomial identities, we obtain the result

Plife(mi;mi′|ji)=Passt(mi;mi′|ki)Pdev(mi+mi′|ji)=(Nm1,…mω)∏i=1g(qjiN)mi(1-q+qjωN)mω×(Nm1′,…mω′)∏i=1g(qjiN)mi′(1-q+qjωN)mω′ (30)

The way this probability factors means that the type of one vesicle offspring is independent of the type of the other offspring given the initial protocell composition and that both follow a Wright-Fisher process. This does not mean that the two daughter protocells are independent after development.

Our population is comprised of a number of non-interacting protocells each of which undergoes a lifecycle as described above. To describe this population, we introduce the summary statistic *F*(*j*_*i*_) which is the frequency in the population of protocells which began their development process with *j*_*i*_ copies of the *i*-th sequence. Let *W*(*j*_*i*_) denote the survivability of a typical protocell which begins its development process with *j*_*i*_ copies of the *i*-th sequence. Such a population may be modelled either as a branching process to describe a growing population or as a Moran process to describe a population which has reached its carrying capacity. In the limit of large population size, the dynamics of either of these models become deterministic and the equilibrium frequencies of both models are determined by the same eigenvalue equation

$$\begin{array}{c} \sum_{j_{i}|\sum_{i}j_{i}=N}K\left(m_{i}|j_{i}\right)F\left(j_{i}\right)=\bar{W}F\left(m_{i}\right) \end{array}$$ (31)

where

K(mi|ji)=W(mi)(Nm1,…mω)∏i=1g(qjiN)mi(1-q+qjωN)mω (32)

and $\bar{W}$ is the mean fitness. Since all the components of the matrix *K*(*m*_*i*_|*j*_*i*_) are non-negative, this equation may be solved numerically by using Perron-Frobenius iteration for small ploidy values (up to 100) and few sequence types (up to three). For higher ploidy values, both the Perron-Frobenius iteration or the Moran Process become cumbersome, so an agent based simulation which also approaches the same limit for large population size was used instead. As in [24], the agent based model algorithm starts with an homogenous populations of protocells with the same number of sequences for each catalytic type. The entire population undergoes development and assortment doubling the number of protocells. Half of the population is selected without replacement with a probability proportional to its fitness. We found that 5000 generations were sufficient to reach the stationary behavior of the model in all the cases studied. Comparing instances the Perron-Frobenius iteration and the agent-based model available shows that even for small population sizes of 1000, the results are already in reasonable agreement with the infinite population limit. Using these values of 5000 generations of 1000 individuals, the computation proceeds sufficiently rapidly that it becomes feasible to scan across ranges of parameter values and generate plots which show how observables vary across parameter space. The model was implemented in Julia version 0.3.11 [49] and the plots were generated in python 2.7. The code used for the simulations can be found in GitHub as: https://github.com/jopejor/protocell_metabolism.

## Supporting information

S1 Fig Average mean metabolic flux for the stationary protocell population of the recycler example. The figure shows the dependence of the average mean metabolic flux of the stationary population with the ploidy and the replication accuracy for the case in Fig 1.
(PDF)

S2 Fig Average mean metabolic flux for the stationary protocell population for the two catalyst architectures. The figure shows the dependence of the average mean metabolic flux of the stationary population with the ploidy for the case in Fig 2.
(PDF)

S3 Fig Average defective type frequency of the stationary protocell population of the recycler example. The curves show the dependence of the average defective type frequency in the stationary population with the ploidy and the replication accuracy for the case in Fig 1.
(PDF)

S1 Text Networks and metabolic fluxes. Chemical reactions, network diagrams and fluxes for each of the networks studied in this work.
(PDF)

## References

[1] Gilbert W. Origin of life: The RNA world. Nature. 1986;319(6055):618–618. 10.1038/319618a0

[2] Robertson MP, Joyce GF. The origins of the RNA world. Cold Spring Harb Perspect Biol. 2012;4(5):1–22. 10.1101/cshperspect.a003608 PMC3331698 20739415

[3] Kun Á, Szilágyi A, Könnyű B, Boza G, Zachar I, Szathmáry E. The dynamics of the RNA world: insights and challenges. Ann N Y Acad Sci. 2015; p. n/a–n/a. 10.1111/nyas.12700 25735569

[4] Ruiz-Mirazo K, Briones C, de la Escosura A. Prebiotic systems chemistry: new perspectives for the origins of life. Chem Rev. 2014;114(1):285–366. 10.1021/cr2004844 24171674

[5] Sutherland JD. The Origin of Life-Out of the Blue. Angew Chem Int Ed. 2016;55:104–121. 10.1002/anie.201506585 26510485

[6] Krupkin M, Matzov D, Tang H, Metz M, Kalaora R, Belousoff MJ, et al A vestige of a prebiotic bonding machine is functioning within the contemporary ribosome. Philos Trans R Soc Lond B Biol Sci. 2011;366(1580):2972–8. 10.1098/rstb.2011.0146 21930590 PMC3158926

[7] Eigen M. Selforganization of matter and the evolution of biological macromolecules. Naturwissenschaften. 1971;58(10):465–523. 10.1007/BF00623322 4942363

[8] Eigen M, Mccaskill J. Molecular Quasi-Speciest. 1988;(1):6881–6891.

[9] Eigen M, Schuster P. The Hypercycle. Naturwissenschaften. 1978;65(7):341–369. 10.1007/BF00439699

[10] Mitteldorf J, Wilson DS. Population viscosity and the evolution of altruism. J Theor Biol. 2000;204(4):481–96. 10.1006/jtbi.2000.2007 10833350

[11] Boerlijst MC, Hogeweg P. Spatial gradients enhance persistence of hypercycles. Phys D Nonlinear Phenom. 1995;88(1):29–39. 10.1016/0167-2789(95)00178-7

[12] Franchi M, Ferris JP, Gallori E. Cations as mediators of the adsorption of nucleic acids on clay surfaces in prebiotic environments. Orig Life Evol Biosph. 2003;33(1):1–16. 10.1023/A:1023982008714 12967270

[13] Nowak Ma. Five rules for the evolution of cooperation. Science. 2006;314(5805):1560–3. 10.1126/science.1133755 17158317 PMC3279745

[14] Traulsen A, Nowak Ma. Evolution of cooperation by multilevel selection. PNAS. 2006;103(29):10952–5. 10.1073/pnas.0602530103 16829575 PMC1544155

[15] Hogeweg P, Takeuchi N. Multilevel selection in models of prebiotic evolution: compartments and spatial self-organization. Orig Life Evol Biosph. 2003;33(4-5):375–403. 10.1023/A:1025754907141 14604183

[16] Solé RV, Munteanu A, Rodriguez-Caso C, Macía J. Synthetic protocell biology: from reproduction to computation. Philos Trans R Soc Lond B Biol Sci. 2007;362(1486):1727–39. 10.1098/rstb.2007.2065 17472932 PMC2442389

[17] Niesert U, Harnasch D, Bresch C. Origin of life between Scylla and Charybdis. J Mol Evol. 1981;17(6):348–53. 10.1007/BF01734356 7288889

[18] Silvestre D, Fontanari J. Preservation of information in a prebiotic package model. Phys Rev E. 2007;75(5):051909 10.1103/PhysRevE.75.051909 17677100

[19] Szathmáry E, Demeter L. Group selection of early replicators and the origin of life. J Theor Biol. 1987;128(4):463–86. 10.1016/S0022-5193(87)80191-1 2451771

[20] Grey D, Hutson V, Szathmary E. A Re-Examination of the Stochastic Corrector Model. Proc R Soc B Biol Sci. 1995;262(1363):29–35. 10.1098/rspb.1995.0172

[21] Zintzaras E, Santos M, Szathmáry E. Selfishness versus functional cooperation in a stochastic protocell model. J Theor Biol. 2010;267(4):605–13. 10.1016/j.jtbi.2010.09.011 20837027

[22] Könnyu B, Czárán T. The evolution of enzyme specificity in the metabolic replicator model of prebiotic evolution. PLoS One. 2011;6(6):e20931 10.1371/journal.pone.0020931 21698204 PMC3116859

[23] Santos M, Zintzaras E, Szathmáry E. Origin of sex revisited. Orig Life Evol Biosph. 2003;33(4-5):405–32. 10.1023/A:1025759024888 14604184

[24] Santos M, Zintzaras E, Szathmáry E. Recombination in primeval genomes: a step forward but still a long leap from maintaining a sizable genome. J Mol Evol. 2004;59(4):507–19. 10.1007/s00239-004-2642-7 15638462

[25] Szilágyi A, Kun Á, Szathmáry E. Early evolution of efficient enzymes and genome organization; 2012. 10.1186/1745-6150-7-38 PMC3534232 23114029

[26] Mavelli F. Stochastic simulations of minimal cells: the Ribocell model. BMC Bioinformatics. 2012;13(Suppl 4):S10 10.1186/1471-2105-13-S4-S10 22536956 PMC3303737

[27] Kacser H, Burns JA. The molecular basis of dominance. Genetics. 1981;97(3-4):639–666. 7297851 10.1093/genetics/97.3-4.639 PMC1214416

[28] Bergman A, Siegal ML. Evolutionary capacitance as a general feature of complex gene networks. Nature. 2003;424(6948):549–52. 10.1038/nature01765 12891357

[29] Gunawardena J. Chemical reaction network theory for in-silico biologists; 2003.

[30] Orth JD, Thiele I, Palsson BØ. What is flux balance analysis? Nat Biotechnol. 2010;28(3):245–8. 10.1038/nbt.1614 20212490 PMC3108565

[31] Fontanari JF, Serva M. Solvable model for template coexistence in protocells. EPL (Europhysics Lett. 2013;101(3):38006 10.1209/0295-5075/101/38006

[32] Fontanari JF, Santos M, Szathmáry E. Coexistence and error propagation in pre-biotic vesicle models: a group selection approach. J Theor Biol. 2006;239(2):247–56. 10.1016/j.jtbi.2005.08.039 16243358

[33] Hubai AG, Kun A. Maximal gene number maintainable by stochastic correction—The second error threshold. J Theor Biol. 2016;405:29–35. 10.1016/j.jtbi.2016.02.007 26876752

[34] Zhao J, Yu H, Luo JH, Cao ZW, Li YX. Hierarchical modularity of nested bow-ties in metabolic networks. BMC Bioinformatics. 2006;7:386 10.1186/1471-2105-7-386 16916470 PMC1560398

[35] Corominas-Murtra B, Goñi J, Solé RV, Rodríguez-Caso C. On the origins of hierarchy in complex networks. PNAS. 2013;110(33):13316–21. 10.1073/pnas.1300832110 23898177 PMC3746874

[36] Szathmáry E. Toward major evolutionary transitions theory 2.0. Proc Natl Acad Sci. 2015;(14):201421398. 10.1073/pnas.1421398112 PMC4547294 25838283

[37] Boza G, Szilágyi A, Kun Á, Santos M, Szathmáry E. Evolution of the Division of Labor between Genes and Enzymes in the RNA World. PLoS Comput Biol. 2014;10(12):e1003936 10.1371/journal.pcbi.1003936 25474573 PMC4256009

[38] Smith JM, Szathmáry E. The origin of chromosomes. I. Selection for linkage. J Theor Biol. 1993;164(4):437–46. 10.1006/jtbi.1993.1165 8264246

[39] Kacser H, Beeby R. Evolution of catalytic proteins. J Mol Evol. 1984;20(1):38–51. 10.1007/BF02101984 6429341

[40] Takeuchi N, Kaneko K, Hogeweg P. Evolutionarily stable disequilibrium: endless dynamics of evolution in a stationary population. Proc R Soc B Biol Sci. 2016;283(1830):20153109 10.1098/rspb.2015.3109 PMC4874703 27147095

[41] Karr JR, Sanghvi JC, Macklin DN, Gutschow MV, Jacobs JM, Bolival B, et al A Whole-Cell Computational Model Predicts Phenotype from Genotype. Cell. 2012;150(2):389–401. 10.1016/j.cell.2012.05.044 22817898 PMC3413483

[42] Bianconi G, Zhao K, Chen Ia, Nowak Ma. Selection for replicases in protocells. PLoS Comput Biol. 2013;9(5):e1003051 10.1371/journal.pcbi.1003051 23671413 PMC3649988

[43] Markvoort AJ, Sinai S, Nowak Ma. Computer simulations of cellular group selection reveal mechanism for sustaining cooperation. J Theor Biol. 2014; p. 1–11. 10.1016/j.jtbi.2014.04.029 24799131

[44] Mori M, Ponce-de Leon M, Peretó J, Montero F. Metabolic complementation in bacterial communities: Necessary conditions and optimality. Front Microbiol. 2016;7(OCT):1–14. 27774085 10.3389/fmicb.2016.01553 PMC5054487

[45] Ichihashi N, Usui K, Kazuta Y, Sunami T, Matsuura T, Yomo T. Darwinian evolution in a translation-coupled RNA replication system within a cell-like compartment. Nat Commun. 2013;4:2494 10.1038/ncomms3494 24088711

[46] Matsumura S, Kun Á, Ryckelynck M, Coldren F, Szilágyi A, Jossinet F, et al Transient compartmentalization of RNA replicators prevents extinction due to parasites. Science. 2016;354(6317):1293–1296. 10.1126/science.aag1582 27940874

[47] Alon U. Network motifs: theory and experimental approaches. Nat Rev Genet. 2007;8(6):450–61. 10.1038/nrg2102 17510665

[48] Kaneko K, Yomo T. On a kinetic origin of heredity: minority control in a replicating system with mutually catalytic molecules. J Theor Biol. 2002;214:563–576. 10.1006/jtbi.2001.2481 11851368

[49] Bezanson J, Karpinski S, Shah VB, Edelman A. Julia: A Fast Dynamic Language for Technical Computing. arXiv:12095145. 2012; p. 1–27.

