# Supplementary figures and images for: The evolutionary dynamics of metabolic protocells
Source: PLoS Comput Biol. 2018 Jul 20;14(7):e1006265. doi: 10.1371/journal.pcbi.1006265 (PMC6070278; doi:10.1371/journal.pcbi.1006265)

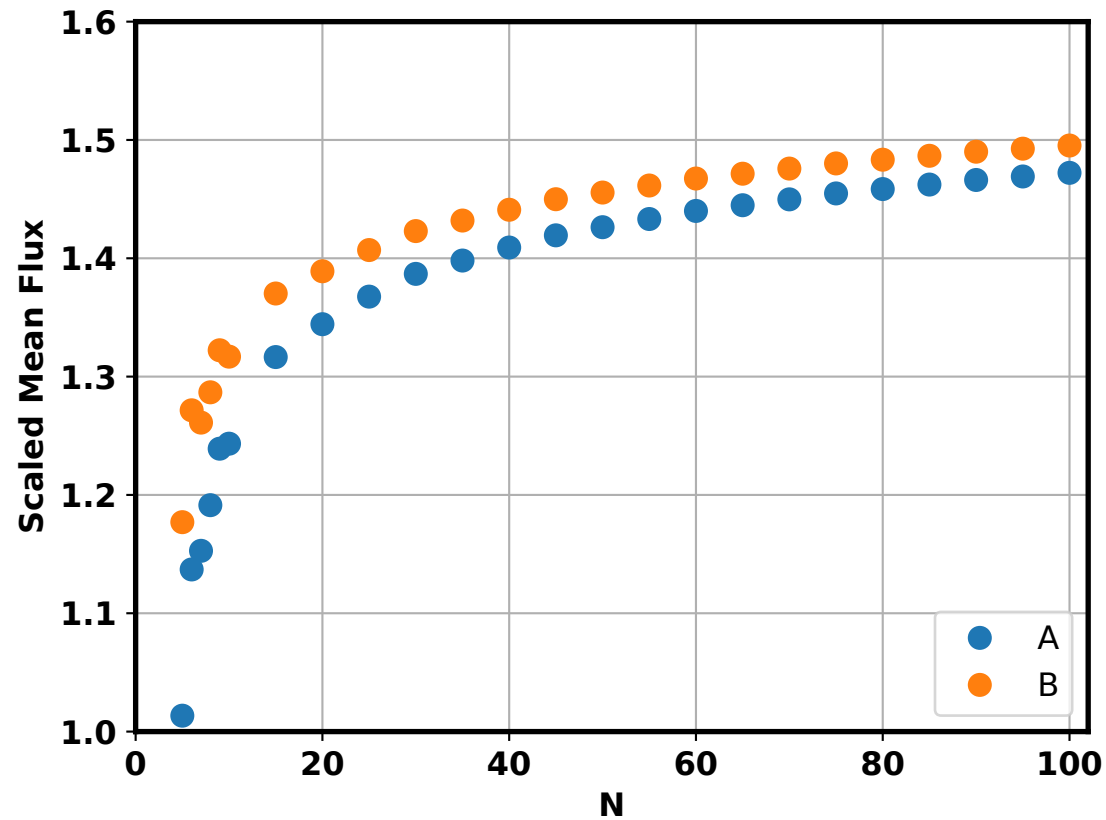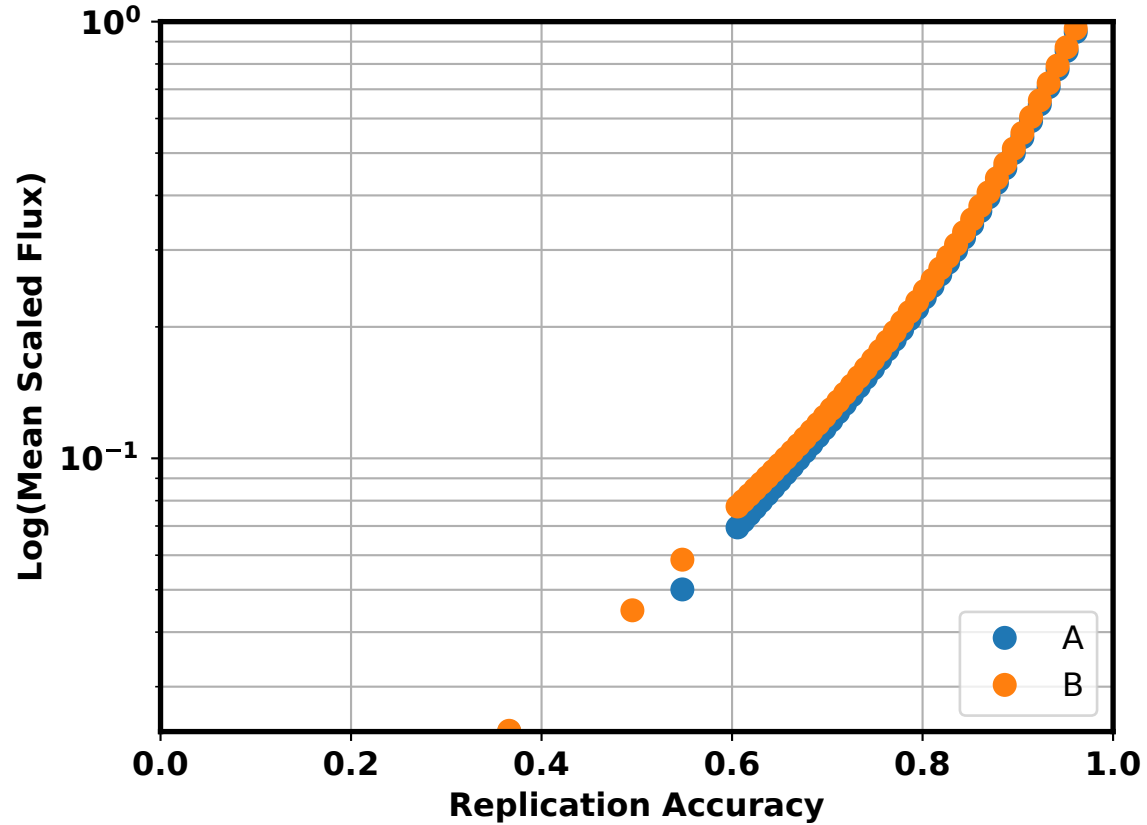

Supplement: S1 Fig — The figure shows the dependence of the average mean metabolic flux of the stationary population with the ploidy and the replication accuracy for the case in Fig 1. (PDF) [file pcbi.1006265.s001.pdf]

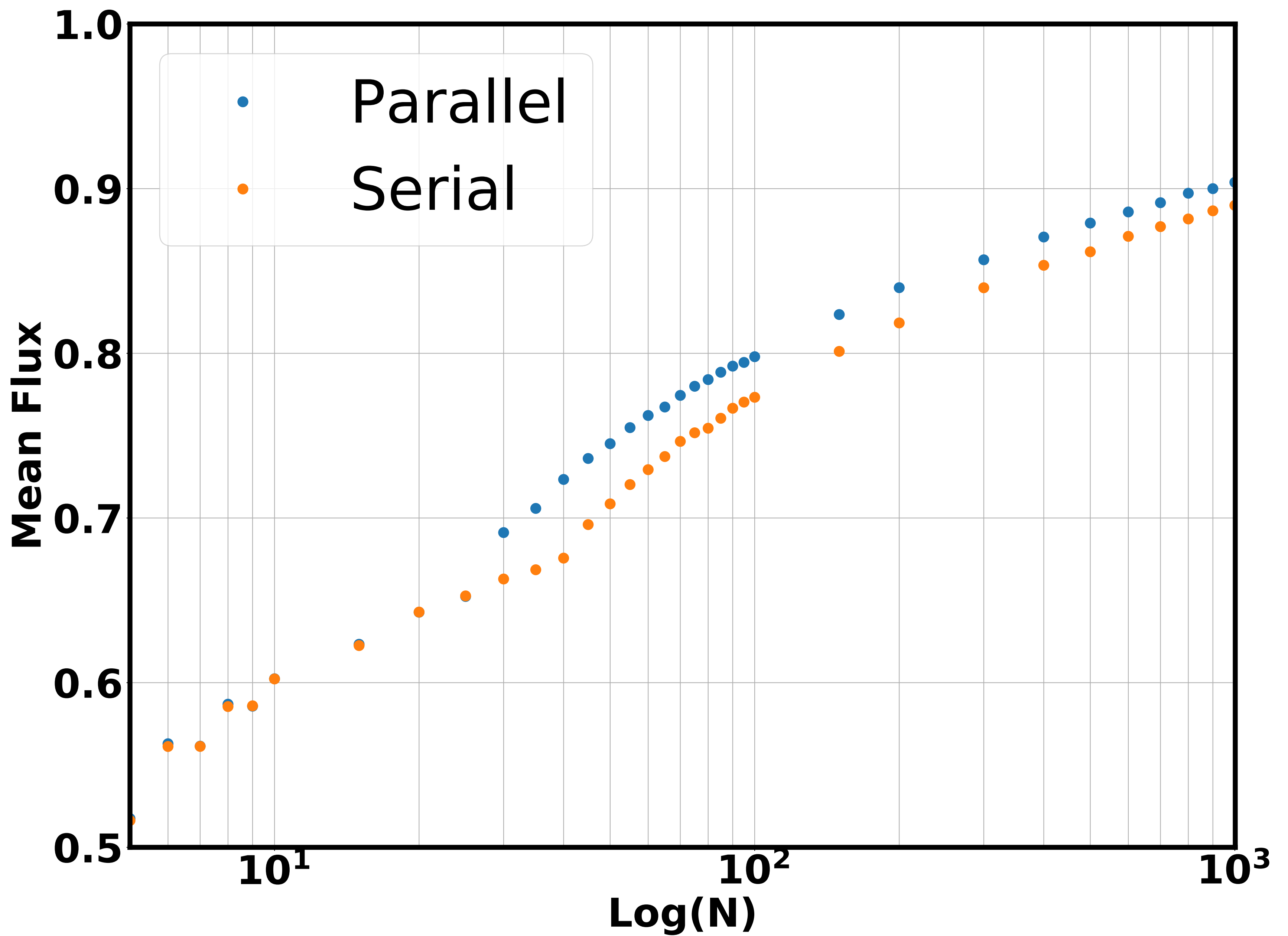

Supplement: S2 Fig — The figure shows the dependence of the average mean metabolic flux of the stationary population with the ploidy for the case in Fig 2. (PDF) [file pcbi.1006265.s002.pdf]

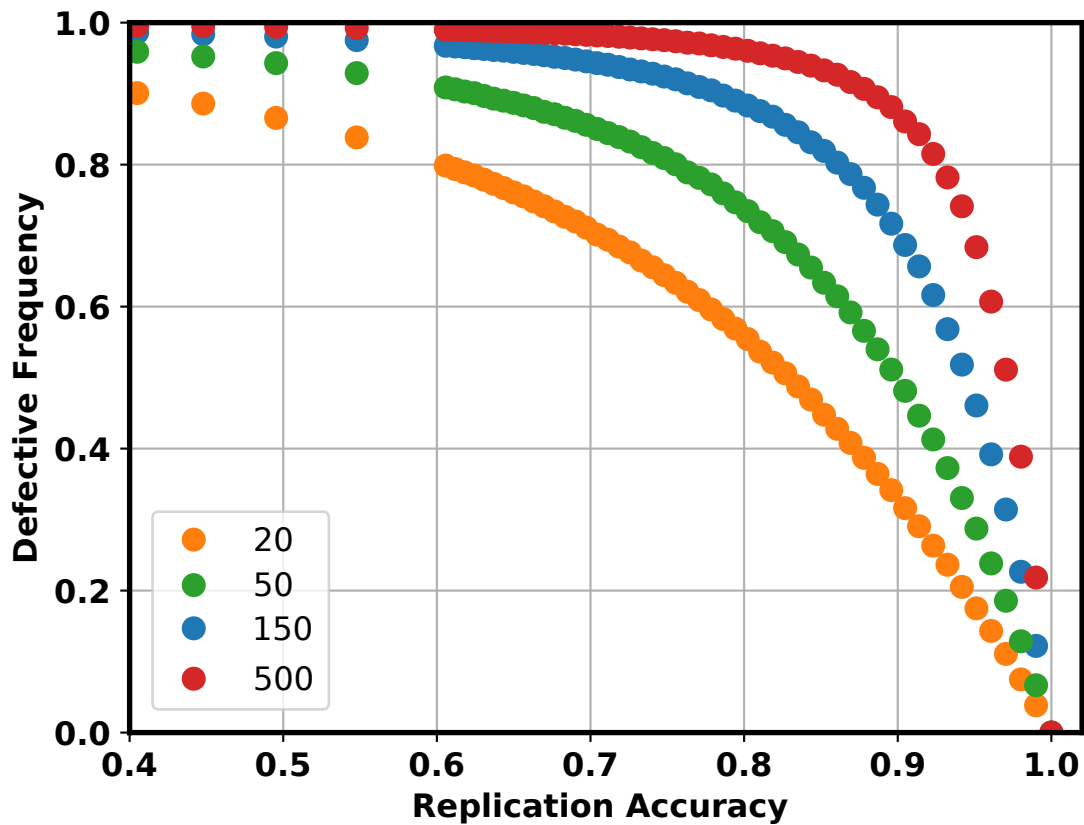

Supplement: S3 Fig — The curves show the dependence of the average defective type frequency in the stationary population with the ploidy and the replication accuracy for the case in Fig 1. (PDF) [file pcbi.1006265.s003.pdf]
