# Supplementary material for: The evolutionary dynamics of metabolic protocells
Source: PLoS Comput Biol. 2018 Jul 20;14(7):e1006265. doi: 10.1371/journal.pcbi.1006265 (PMC6070278; doi:10.1371/journal.pcbi.1006265)
Supplement: S1 Text — Chemical reactions, network diagrams and fluxes for each of the networks studied in this work. (PDF) [file pcbi.1006265.s004.pdf]

Supporting Information

Derivation of fluxes for three catalyst networks

- **Serial**

The serial recycler case reactions:

$$\begin{aligned} A + \alpha &\rightarrow c_M^\alpha M + W1 \\ W_1 + \beta &\rightarrow \beta + c_M^\beta M + W_2 \\ W_1 &\rightarrow \emptyset \\ W_2 + \gamma &\rightarrow \gamma + c_M^\gamma M \\ W_2 &\rightarrow \emptyset \end{aligned} \tag{33}$$

(34)

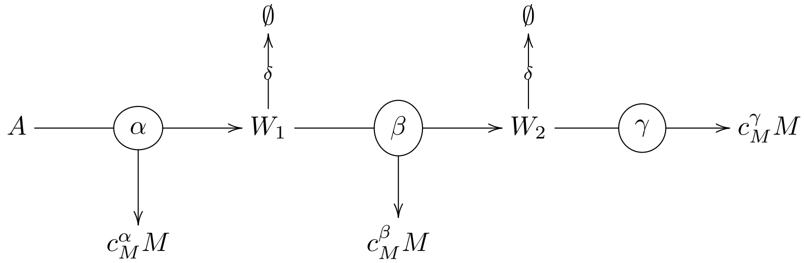

The corresponding flux:

$$\Phi_{Serial} = k_{\text{cat}}^\alpha[\alpha] + c_M^\beta \min(k_{\text{cat}}^\alpha[\alpha], k_{\text{cat}}^\beta[\beta]) + c_M^\gamma \min(k_{\text{cat}}^\alpha[\alpha], k_{\text{cat}}^\beta[\beta], k_{\text{cat}}^\gamma[\gamma]) \tag{35}$$

- **Parallel**

For the parallel recycler case we have the following reactions:

$$\begin{aligned} A + \alpha &\rightarrow c_M^\alpha M + \alpha + W_1 + W_2 \\ W_1 + \beta &\rightarrow \beta + c_M^\beta M \\ w_1 &\rightarrow \emptyset \\ W_2 + \gamma &\rightarrow \gamma + c_M^\gamma M \\ W_2 &\rightarrow \emptyset \end{aligned} \tag{36}$$

(37)

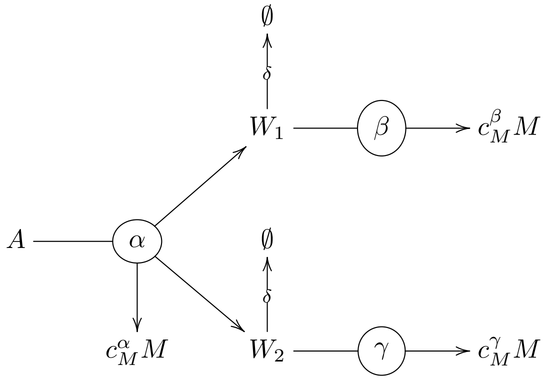

The corresponding flux:

$$\Phi_{Parallel} = k_{\text{cat}}^\alpha[\alpha] + c_M^\beta \min(k_{\text{cat}}^\alpha[\alpha], k_{\text{cat}}^\beta[\beta]) + c_M^\gamma \min(k_{\text{cat}}^\alpha[\alpha], k_{\text{cat}}^\gamma[\gamma]) \tag{38}$$

Derivation of fluxes for the bimolecular motiff

For the bimolecular architecture we have the following reactions:

$$\begin{aligned} A + \alpha &\rightarrow \alpha + c_M^\alpha M + W_1 \\ A + \beta &\rightarrow \beta + c_M^\beta M + W_2 \\ W_1 + W_2 + \gamma &\rightarrow c_M^\gamma M \\ W_1 &\rightarrow \emptyset \\ W_2 &\rightarrow \emptyset \end{aligned} \tag{39}$$

(40)

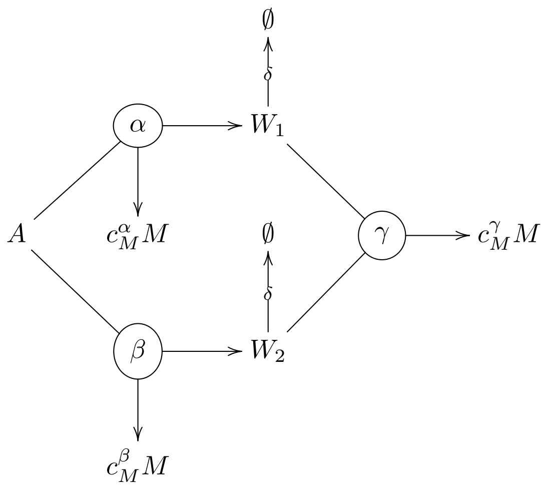

For this architecture, the velocities of the first two reactions are:

$$v_\alpha = k_{\text{cat}}^\alpha[\alpha] \tag{41}$$

$$v_\beta = k_{\text{cat}}^\beta[\beta] \tag{42}$$

and the velocity of the third reaction depends upon them:

$$v_\gamma = \min\left(v_\alpha, v_\beta, k_{\text{cat}}^\gamma[\gamma]\right) = \min\left(k_{\text{cat}}^\alpha[\alpha], k_{\text{cat}}^\beta[\beta], k_{\text{cat}}^\gamma[\gamma]\right) \tag{43}$$

Hence the total flux equals:

$$\Phi_{Bimolecular} = c_M^\alpha k_{\text{cat}}^\alpha[\alpha] + c_M^\beta k_{\text{cat}}^\beta[\beta] + c_M^\gamma \min\left(k_{\text{cat}}^\alpha[\alpha], k_{\text{cat}}^\beta[\beta], k_{\text{cat}}^\gamma[\gamma]\right) \tag{44}$$

Derivation of the fluxes for the four catalyst networks

We will now derive the metabolite fluxes of the following four catalyst architectures:

- **Serial**

For the four catalyst serial recycler the following reactions are considered:

$$
\begin{aligned}
A + \alpha &\to \alpha + W_1 + c_M^{\alpha} M \\
W_1 + \beta &\to \beta + W_2 + c_M^{\beta} M \\
W_1 &\to \emptyset \\
W_2 + \gamma &\to \gamma + W_3 + c_M^{\gamma} M \\
W_2 &\to \emptyset \\
W_3 + \theta &\to \theta + c_M^{\theta} M \\
W_3 &\to \emptyset
\end{aligned} \tag{45}
$$

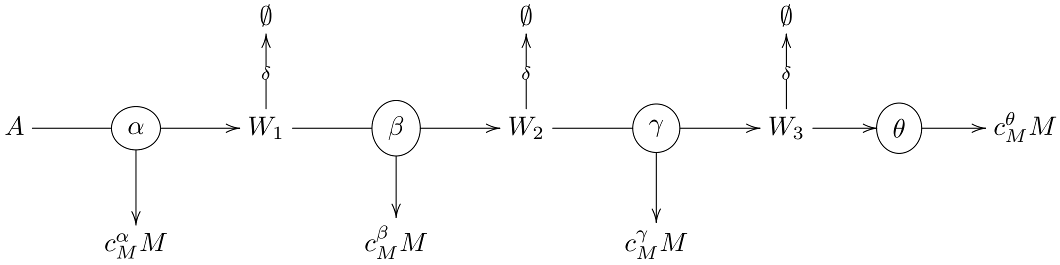

(46)

The corresponding flux:

$$
\begin{aligned}
\Phi_{\text{Serial}} = c_M^{\alpha} k_{\text{cat}}^{\alpha}[\alpha] + c_M^{\beta} \min(k_{\text{cat}}^{\alpha}[\alpha], k_{\text{cat}}^{\beta}[\beta]) + c_M^{\gamma} \min(k_{\text{cat}}^{\alpha}[\alpha], k_{\text{cat}}^{\beta}[\beta], k_{\text{cat}}^{\gamma}[\gamma]) \\
+ c_M^{\theta} \min(k_{\text{cat}}^{\alpha}[\alpha], k_{\text{cat}}^{\beta}[\beta], k_{\text{cat}}^{\gamma}[\gamma], k_{\text{cat}}^{\theta}[\theta])
\end{aligned} \tag{47}
$$

- **Parallel**

The reactions:

$$
\begin{aligned}
A + \alpha &\to \alpha + W_1 + W_2 + W_3 + c_M^{\alpha} M \\
W_1 + \beta &\to \beta + c_M^{\beta} M \\
W_1 &\to \emptyset \\
W_2 + \gamma &\to \gamma + c_M^{\gamma} M \\
W_2 &\to \emptyset \\
W_3 + \theta &\to \theta + c_M^{\theta} M \\
W_3 &\to \emptyset
\end{aligned} \tag{48}
$$

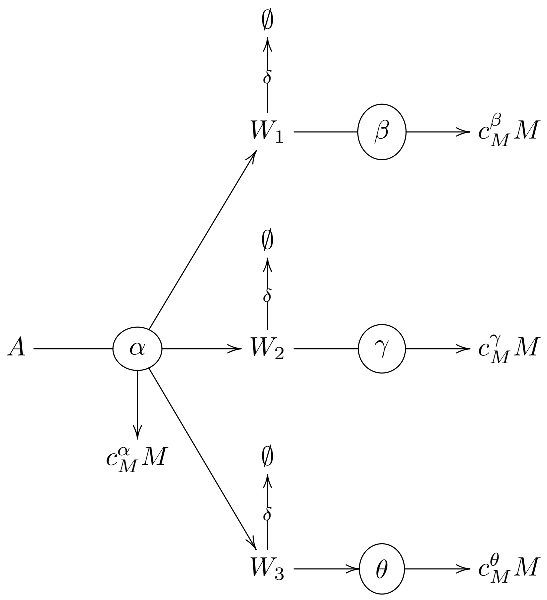

The corresponding flux:

$$\Phi_{\text{parallel}} = c_M^\alpha k_{\text{cat}}^\alpha[\alpha] + c_M^\beta \min(k_{\text{cat}}^\alpha[\alpha], k_{\text{cat}}^\beta[\beta]) + c_M^\gamma \min(k_{\text{cat}}^\alpha[\alpha], k_{\text{cat}}^\gamma[\gamma]) + c_M^\theta \min(k_{\text{cat}}^\alpha[\theta], k_{\text{cat}}^\theta[\theta] \tag{49}$$

- **Parallel-Serial**

The reactions:

$$\begin{aligned} A + \alpha &\to \alpha + W_1 + W_2 + c_M^\alpha M \\ W_1 + \beta &\to \beta + W_3 + c_M^\beta M \\ W_1 &\to \emptyset \\ W_2 + \gamma &\to \gamma + c_M^\gamma M \\ W_2 &\to \emptyset \\ W_3 + \theta &\to \theta + c_M^\theta M \\ W_3 &\to \emptyset \end{aligned} \tag{50}$$

(51)

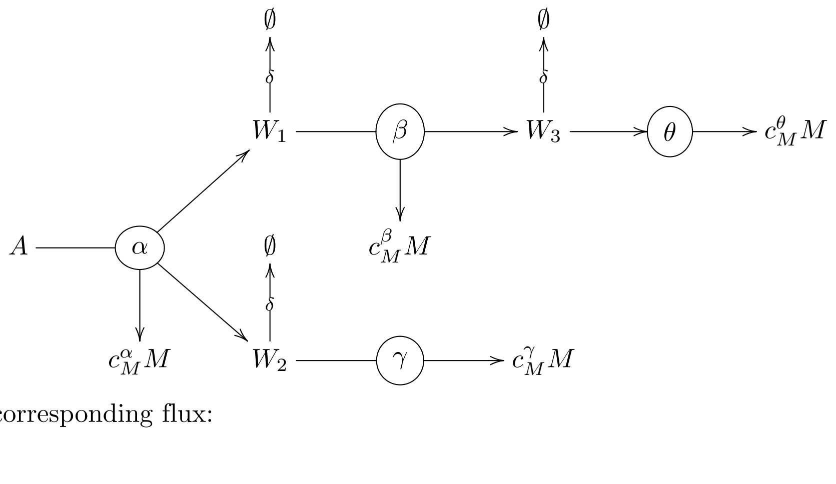

The corresponding flux:

$$\Phi_{\text{parallel-serial}} = c_M^\alpha k_{\text{cat}}^\alpha[\alpha] + c_M^\beta \min(k_{\text{cat}}^\alpha[\alpha], k_{\text{cat}}^\beta[\beta]) + c_M^\gamma \min(k_{\text{cat}}^\alpha[\alpha], k_{\text{cat}}^\gamma[\gamma]) + c_M^\theta \min(k_{\text{cat}}^\alpha[\alpha], k_{\text{cat}}^\beta[\beta], k_{\text{cat}}^\theta[\theta]) \tag{52}$$

- **Serial-Parallel**

The reactions:

$$\begin{aligned} A + \alpha &\to \alpha + W_1 + c_M^\alpha M \\ W_1 + \beta &\to \beta + W_2 + W_3 + c_M^\beta M \\ W_1 &\to \emptyset \\ W_2 + \gamma &\to \gamma + c_M^\gamma M \\ W_2 &\to \emptyset \\ W_3 + \theta &\to \theta + c_M^\theta M \\ W_3 &\to \emptyset \end{aligned} \tag{53}$$

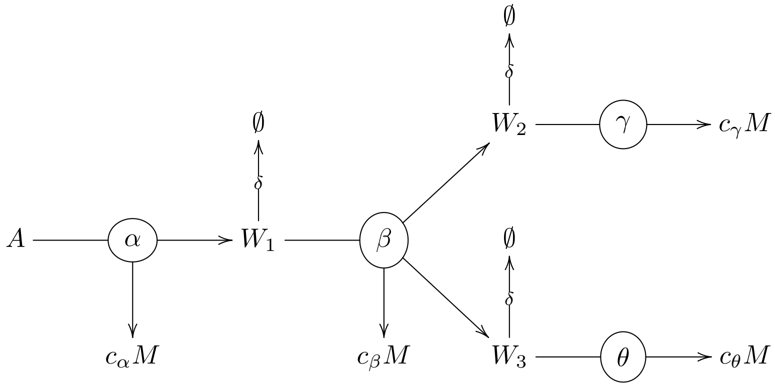

) (54)

The corresponding flux:

$$\Phi_{\text{serial-parallel}} = c_M^\alpha k_{\text{cat}}^\alpha[\alpha] + c_M^\beta \min(k_{\text{cat}}^\alpha[\alpha], k_{\text{cat}}^\beta[\beta]) + c_M^\gamma \min(k_{\text{cat}}^\alpha[\alpha], k_{\text{cat}}^\beta[\beta], k_{\text{cat}}^\gamma[\gamma]) + c_M^\theta \min(k_{\text{cat}}^\alpha[\alpha], k_{\text{cat}}^\beta[\beta], k_{\text{cat}}^\theta[\theta]) \tag{55}$$

- **Bimolecular**

The reactions:

$$\begin{aligned} A + \alpha &\to \alpha + W_1 + W_2 + c_M^\alpha M \\ W_1 + \beta &\to W_3 + c_M^\beta M \\ W_1 &\to \emptyset \\ W_2 + \gamma &\to \gamma + W_4 + c_M^\gamma M \\ W_2 &\to \emptyset \\ W_3 + W_4 + \theta &\to \theta + c_M^\theta M \\ W_3 &\to \emptyset \\ W_4 &\to \emptyset \end{aligned} \tag{56}$$

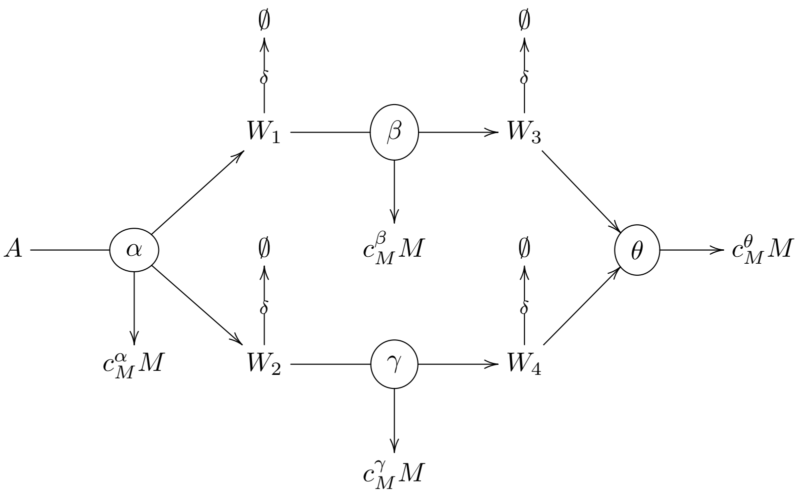

The corresponding flux:

$$\Phi_{\text{Bimolecular}} = c_M^{\alpha} k_{\text{cat}}^{\alpha}[\alpha] + c_M^{\beta} \min(k_{\text{cat}}^{\alpha}[\alpha], k_{\text{cat}}^{\beta}[\beta]) + c_M^{\gamma} \min(k_{\text{cat}}^{\alpha}[\alpha], k_{\text{cat}}^{\gamma}[\gamma]) \\ + c_M^{\theta} \min(k_{\text{cat}}^{\alpha}[\alpha], k_{\text{cat}}^{\beta}[\beta], k_{\text{cat}}^{\gamma}[\gamma], k_{\text{cat}}^{\theta}[\theta]) \quad (57)$$
